# Draft genome sequence of *Escherichia coli* MTR_GS_S1457 strain isolated from a soil sample of a vegetable garden in Gazipur, Bangladesh

**DOI:** 10.1128/mra.00021-24

**Published:** 2024-05-17

**Authors:** Md. Saiful Islam, Pritom Kumar Pramanik, Md. Liton Rana, Md. Ashek Ullah, Fahim Haque Neloy, Srinivasan Ramasamy, Pepijn Schreinemachers, Ricardo Oliva, Md. Tanvir Rahman

**Affiliations:** 1 Department of Microbiology and Hygiene, Faculty of Veterinary Science, Bangladesh Agricultural University, Mymensingh, Bangladesh; 2 Department of Animal Sciences, University of California—Davis, Davis, California, USA; 3 Safe and Sustainable Value Chains Flagship Program, World Vegetable Center, Shanhua, Tainan, Taiwan; 4 Healthy Diets and Enabling Impact Flagship Programs, World Vegetable Center–East and Southeast Asia, Bangkok, Thailand; University of Strathclyde, Glasgow, United Kingdom

**Keywords:** whole-genome sequencing, *Escherichia coli*, soil, environment, vegetable gardening system, Bangladesh

## Abstract

We announce the sequence of the *Escherichia coli* MTR_GS_S1457 strain isolated from a soil sample of a vegetable gardening system for the first time in Bangladesh. With a length of 4,918,647 bp, this strain contained one plasmid, two CRISPR arrays, 54 predicted antibiotic resistance genes, and 81 predicted virulence factor genes.

## ANNOUNCEMENT

Food safety hinges on bacteria transmission from contaminated soil to edible plant parts, including *Escherichia coli* strains (1). While not all strains harm humans, enteropathogenic or enterohemorrhagic strains pose health risks (2). Manure used as fertilizer in family gardens could harbor such strains and potentially be transferred to humans (3). This first study in Bangladesh analyzes genome-based *E. coli* from soil in a family vegetable garden.

In November 2022, 2 grams of soil was aseptically collected from a family vegetable garden in the Gazipur district of Bangladesh (25.6135°N, 83.5070°E) by penetrating the soil surface (0–5 cm depth, 2.5 cm diameter). The soil was then homogenized and sieved using a 6.35 mm mesh sieve. The sample was then introduced into the nutrient broth (HiMedia, India; catalog number: M002-500G), incubated at 37°C for 24 hours, spread onto an eosin methylene blue agar plate, and the plate was then incubated overnight at 37°C. The colonies, showing metallic green sheens on the plate, underwent Gram staining and biochemical tests (indole and oxidase) to isolate *E. coli* (4). The *E. coli* MTR_GS_S1457 strain was cultured in nutrient broth aerobically overnight at 37°C, and the DNA extraction from the cultured broth was done using the DNeasy Blood and Tissue Kit (Qiagen, Hilden, Germany). Subsequently, the DNA library was prepared with the Nextera DNA Flex Library Prep Kit (Illumina, San Diego, CA, USA). Genome sequencing was conducted on the Illumina NextSeq2000 platform, generating paired-end reads with a 2 × 150 bp length. The genome assembly was performed by Unicycler v0.4.9 (5), followed by the trimming of raw paired-end reads (*n* = 2,902,088) using Trimmomatic v0.39 (6) to remove Illumina adapters, known Illumina artifacts, and phiX reads from the data set. Quality assessment was performed through FastQC v0.11.7 (7). Genome annotation was performed using the NCBI Prokaryotic Genome Annotation Pipeline (PGAP v6.6) (8). Sequence type was predicted using MLST v.2.0 (9) using the Center for Genomic Epidemiology database (https://genomicepidemiology.org/services/); serotype by SerotypeFinder v.2.0 (10); the pathogenicity index by PathogenFinder v.1.1 (11); CRISPR arrays and prophages by CRISPRimmunity (12); plasmids by PlasmidFinder v.2.1 (13); antibiotic resistance genes (ARGs) by the Comprehensive Antibiotic Resistance Database (CARD v.3.2.4) with RGI main v.6.0.2 (14); and virulence factor genes (VFGs) by the virulence factor database with VFanalyzer (15). Unless otherwise noted, default parameters were used for all software.

The assembled *E. coli* MTR_GS_S1457 strain comprised 112 contigs, with a guanine-cytosine content of 50.6%. The overall genome size was 4,918,647 bp with a coverage of 69.01×. The *N*_50_ for the assembled contigs was 222,202 bp. This genome had 4,832 genes, 4,749 protein-coding sequences, 83 RNA genes, 2 CRISPR arrays with nine signature genes (*cas2*, *cas1*, *cas6e*, *cas5*, *cas7*, *cse2gr11*, *cas8e*, *cas3*, and *WYL*), 13 prophages, and 1 plasmid. The serotype of the strain was predicted to be O51:H40, a probable enteropathogenic *E. coli*. With a pathogenicity index of 0.938, our genome was typed as ST19, based on eight-gene core-genome multilocus sequence typing (e.g., *dinB*, *icdA*, *pabB*, *polB*, *putP*, *trpA*, *trpB*, and *uidA*). Furthermore, the genome contained 54 predicted ARGs and 81 predicted VFGs.

## Data Availability

The WGS shotgun analysis of *E. coli* MTR_GS_V1777 was deposited to GenBank under the accession number JAVTVQ000000000. The relevant data, including the raw reads, were submitted with BioProject accession number PRJNA1020214, BioSample accession number SAMN37518441, and SRA accession number SRR26151865. In this version, the specific version being referred to is identified as JAVTVQ000000000.1.
